# iSymBase: an integrative functional-genomic platform for ecological exploration of insect symbionts

**DOI:** 10.1093/ismeco/ycag128

**Published:** 2026-05-13

**Authors:** Zuoqi Wang, Yingjie Zhu, Xunyan Liu, Zonghuan Li, Jianyang Bai, Maihao Zou, Chaowei Zhang, Ying Liu, Fei Li, Kang He

**Affiliations:** State Key Laboratory of Rice Biology and Breeding & Ministry of Agricultural and Rural Affairs Key Laboratory of Molecular Biology of Crop Pathogens and Insect Pests & Zhejiang Key Laboratory of Biology and Ecological Regulation of Crop Pathogens and Insects, Institute of Insect Sciences, Zhejiang University, Hangzhou, Zhejiang 310058, China; State Key Laboratory of Rice Biology and Breeding & Ministry of Agricultural and Rural Affairs Key Laboratory of Molecular Biology of Crop Pathogens and Insect Pests & Zhejiang Key Laboratory of Biology and Ecological Regulation of Crop Pathogens and Insects, Institute of Insect Sciences, Zhejiang University, Hangzhou, Zhejiang 310058, China; State Key Laboratory of Rice Biology and Breeding & Ministry of Agricultural and Rural Affairs Key Laboratory of Molecular Biology of Crop Pathogens and Insect Pests & Zhejiang Key Laboratory of Biology and Ecological Regulation of Crop Pathogens and Insects, Institute of Insect Sciences, Zhejiang University, Hangzhou, Zhejiang 310058, China; State Key Laboratory of Rice Biology and Breeding & Ministry of Agricultural and Rural Affairs Key Laboratory of Molecular Biology of Crop Pathogens and Insect Pests & Zhejiang Key Laboratory of Biology and Ecological Regulation of Crop Pathogens and Insects, Institute of Insect Sciences, Zhejiang University, Hangzhou, Zhejiang 310058, China; Anhui Provincial Key Laboratory of Biological Control, Engineering Research Center of Fungal Biotechnology, School of Forestry and Landscape Architecture, Anhui Agricultural University, Hefei, Anhui 230036, China; State Key Laboratory of Rice Biology and Breeding & Ministry of Agricultural and Rural Affairs Key Laboratory of Molecular Biology of Crop Pathogens and Insect Pests & Zhejiang Key Laboratory of Biology and Ecological Regulation of Crop Pathogens and Insects, Institute of Insect Sciences, Zhejiang University, Hangzhou, Zhejiang 310058, China; State Key Laboratory of Rice Biology and Breeding & Ministry of Agricultural and Rural Affairs Key Laboratory of Molecular Biology of Crop Pathogens and Insect Pests & Zhejiang Key Laboratory of Biology and Ecological Regulation of Crop Pathogens and Insects, Institute of Insect Sciences, Zhejiang University, Hangzhou, Zhejiang 310058, China; Key Laboratory of Green Prevention and Control of Agricultural Transboundary Pests of Yunnan Province/Agricultural Environment and Resource Research Institute, Yunnan Academy of Agricultural Sciences, Kunming, Yunnan 650205, China; State Key Laboratory of Rice Biology and Breeding & Ministry of Agricultural and Rural Affairs Key Laboratory of Molecular Biology of Crop Pathogens and Insect Pests & Zhejiang Key Laboratory of Biology and Ecological Regulation of Crop Pathogens and Insects, Institute of Insect Sciences, Zhejiang University, Hangzhou, Zhejiang 310058, China; State Key Laboratory of Rice Biology and Breeding & Ministry of Agricultural and Rural Affairs Key Laboratory of Molecular Biology of Crop Pathogens and Insect Pests & Zhejiang Key Laboratory of Biology and Ecological Regulation of Crop Pathogens and Insects, Institute of Insect Sciences, Zhejiang University, Hangzhou, Zhejiang 310058, China

**Keywords:** insect symbionts, database, gut microbiome, endosymbionts, extracellular symbionts

## Abstract

Insect symbionts play essential roles in host biology, influencing nutrition, immunity, reproduction, and environmental adaptation, ultimately shaping insect physiology, ecology, and evolution. With the rapid growth of functional and genomic datasets on insect symbionts, there remains a critical need for a dedicated platform to systematically compile, organize, and analyze these datasets from an integrative ecological perspective. Here, we developed an insect Symbiont database, named as iSymBase, by manually curating functional records and genomic datasets of insect symbionts from published academic literature. Currently, iSymBase contains over 2657 insect symbiont functional records spanning 795 host species, along with 1494 metagenomes, 14 992 amplicon datasets, and standardized genome and gene catalogs, providing a comprehensive resource for ecological and comparative insect symbiont researches. iSymBase offers standardized query functionalities, such as data browsing, keyword associative search, sequence alignment, data download, and submission. Beyond conventional database functionalities, iSymBase provides several innovative tools: insect–symbiont interaction network for host–symbiont ecological relationships, a batch annotation tool for detecting ecologically functional symbionts from microbiome profiles, and an artificial intelligence (AI)-powered chatbot iSymSeek designed to assist researchers with related knowledge queries. Taken together, iSymBase will serve as an open-access and continually updated platform for storing, querying, and analyzing insect symbiont data, supporting ecological exploration of host–symbiont interactions, symbiont functional diversity, and microbiome-driven adaptation.

Database URL: http://symbiont.insect-genome.com/.

## Graphical Abstract

**Graphical Abstract:**
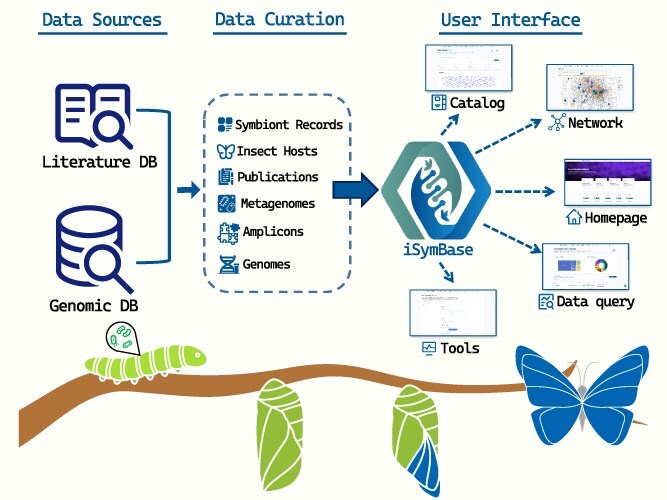

## Introduction

Insects, like many eukaryotic organisms, host a diverse array of microorganisms that can play crucial roles in their biology, resulting in insects being considered multiorganismal entities [1]. These microorganisms can be classified as intracellular or extracellular symbionts based on their location [2–4], either within insect cells or in external habitats like the cuticle, gut, and oral cavity. Due to the widespread distribution and relative stability [5, 6] intracellular symbionts, as known as endosymbionts, have been a primary focus of studies. Their most typical role is to provide metabolic benefits, including essential amino acids, digestive enzymes, and vitamins, thereby enhancing nutrient absorption in nutrient-poor diets [7–9]. Some endosymbionts, such as *Wolbachia*, can manipulate reproduction or fertility in various ways across a broad range of insect species [6, 10–15]. With increasing research on extracellular symbionts, particularly gut symbionts, their diverse functional roles have also been increasingly recognized. Beyond their similar role in nutrient provision [2, 3], extracellular symbionts are vital for defending against environmental stressors, such as resistance to pathogens and parasitoids [16–18], heat tolerance [19], and the detoxification of pesticides or plant secondary metabolites [2, 20]. Furthermore, recent findings highlighted the significant impact of insect symbionts on complex host behaviors, including learning and memory behaviors [21, 22], interspecies competition [23], and dietary specialization [24].

The dramatic progress in sequencing technologies, coupled with a more comprehensive understanding of insect–symbiont interactions, has enhanced the studies of insect symbiont diversity and functional roles [8, 25, 26]. Unfortunately, functional records and genomic data resources are scattered across journals [1, 27]. Researchers have to spend considerable time and effort in collecting records and datasets for their specific study subjects, particularly in studies involving nonmodel species or across multiple species [7, 8, 25, 28].

To address this fragmentation, several domain-specific databases have been developed to support studies of host–microbe symbioses. For example, CIGAF [29] provides long-term ecological occurrence records of trichomycete fungi associated with aquatic insects, SymGenDB [27] focuses on bacterial symbionts across diverse hosts with an emphasis on genomic and metabolic network analyses, and PHI-base [30] curates experimentally validated pathogenicity and virulence phenotypes for multiple pathogens and their hosts, including insects. Although these resources are valuable, they are not specifically designed for insect symbionts. For instance, among the 2328 bacterial genomes curated in SymGenDB, only 169 correspond to insect symbionts, and of the 246 hosts represented in PHI-base, merely 20 are insects. Furthermore, their phenotypic annotations are primarily oriented toward ecological metadata, pathogenicity, virulence, or metabolic potential and are not intended to capture the broad range of insect symbiont functional traits such as nutritional support, detoxification, immunity enhancement, and behavioral modulation (Table 1). These limitations highlight the need for a comprehensive database that specifically targets insect symbionts, integrating functional phenotypes with genomic and ecological data to enable deeper exploration of their roles and interactions.

**Table 1:** Comparison of iSymBase and other symbiosis databases.

Database	Focus	Data content	Phenotypes	Last update
iSymBase	Broad insect symbionts (gut, endosymbionts, extracellular symbionts)	2657 functional records, 795 insect hosts, 1494 metagenomes, 14 992 amplicons, 3648 genomes	Experimentally validated functional phenotypes (nutrition, immunity, stress tolerance, detoxification, etc.)	2025
CIGAF	Trichomycete fungi (gut fungi of aquatic insects)	3120 collection records of trichomycetes, 335 insect hosts	Ecological occurrence data (host, geography site, climate, collection time)	2023
SymGenDB	Symbiotic bacteria across multiple hosts (not insect-specific)	2328 bacterial genomes (including 169 insect symbiont genomes), 498 hosts	Metabolic capacity inferred from genome-scale networks	2020
PHI-base	Multi-species pathogen–host interactions (plants, animals, humans, insects)	9973 genes, 22 415 interactions, 295 pathogens, 246 hosts (including 20 insect hosts)	Pathogenicity/virulence phenotypes (loss/reduced/increased virulence, effector, resistance, etc.)	2025

Here, we present a new insect symbiont database, iSymBase, which offers a one-stop online platform containing manually curated functional records of insect symbionts and related genomic data resources. The database includes experimentally verified functional information on insect symbionts, along with details on their taxonomy, transmission modes, locations, host information, sample collection details, and supporting references. It also compiles insect symbiont genomic resources, including metagenomes, amplicons, and genomes, together with their further analysis results. To the best of our knowledge, iSymBase is the first web resource that systematically provides insect-symbiont interactions information, allowing users to easily browse, query, visualize, and download the functional records and genomic data of insect symbionts. In addition, a range of online tools is available, enabling users to conduct analyses and comparisons of their own datasets via the database platform. iSymBase will be regularly updated, serving as a vital platform for insect symbiont research, enhancing the comprehensive understanding of insect–symbiont interactions and promote new avenues for future research in insect symbionts.

## Materials and methods

### Symbiont record collection and curation

To collect comprehensive and validated functional symbiont records, a systematic search was conducted in the Web of Science, Google Scholar, and PubMed [31] literature database to retrieve related scientific literature published during January 2008 to July 2024. The literature search strategy employed a combination of key terms, including “insect symbiont,” “insect gut community,” “insect endosymbiont,” and “insect microbiome,” as well as other related terms. The search results from different databases were imported into the literature management software Zotero (v7), where duplicate and incomplete entries were removed. After manually excluding article types such as editorials, conference abstracts, and letters, a total of 9560 relevant articles were retained. In the next stage of manual screening, record titles and abstracts were carefully examined to eliminate literature that was clearly unrelated to the insect symbiont topic. The entire record collection process ensured comprehensive coverage of the insect symbiont field. Full texts from the selected literature were further examined to extract three types of data: (i) basic information of the insect symbionts, including symbiont names, taxonomic ranks, hosts, localization, and transmission modes; (ii) experimentally verified functional descriptions of the symbionts; and (iii) documented genomic data, such as amplicons, metagenomes, and genomes, associated with the symbionts. In the extraction process, the principle of retaining the original descriptions was followed, particularly for symbiont names and functional descriptions. The classification of the symbiont records into 42 categories was based on these functional descriptions (Supporting Information Table S1), with reference to previous reviews that established key frameworks for insect symbiont functional roles, including metabolic interaction typologies [8] and host–symbiont dependency gradients [32]. Based on the curated records, the insect–symbiont interaction network was constructed from relationships between host species and symbiont genera, visualized through a force-directed algorithm powered by ECharts.js (v5.6.0).

### Sequencing data collection and processing

We downloaded and reanalyzed metagenome and amplicon sequencing data associated with insect symbionts from the National Center for Biotechnology Information Sequence Read Archive (NCBI SRA) [31], the National Genomics Data Center (NGDC) [33], and the European Bioinformatics Institute (EBI) [34] published during January 2008 to July 2024. In addition, sequencing data from literature were collected during the process of extracting functional symbiont records. After manually screening, the raw sequencing data were downloaded by fastq-dump (v3.1.1) and first subjected to quality control using Fastp (v0.23.4) [35]. On average, 8.2% of raw reads were filtered out during quality control, and the mean read length of the retained sequences was 262 bp.

Taxonomic profiling for metagenomes was carried by the Kraken2 (v2.1.3) workflow [36] with standard parameters, a k-mer based classifier selected for its computational efficiency and accuracy as demonstrated in benchmark studies [37]. Resultant taxonomic distributions were visualized through interactive hierarchical charts generated by KronaTools (v2.8.1). Additionally, the metagenomes were assembled into contigs using Megahit (v1.2.9) [38] with iterative *k*-mer extensions (*k*-min 27, *k*-max 127) optimized for complex microbial communities. Contigs longer than 1000 bp were binned into metagenome-assembled genomes (MAGs) using MetaBAT2 (v2.17) [39], which integrates sequence composition and coverage information. Contig depth profiles were generated with jgi_summarize_bam_contig_depths (minimum identity 97%) from Bowtie2-aligned BAM files. MetaBAT2 was run with default parameters (--minContig 2500, --maxP 95, --minS 60, --maxEdges 200), and --maxEdges was increased to 500 for complex communities to enhance binning resolution. The quality of MAGs was assessed through CheckM (v2.17) [40] using lineage-specific marker gene sets to evaluate completeness and contamination levels.

As for amplicon sequencing data, preprocessing involved quality control and merging with Fastp (v0.23.4) [35] using the --detect_adapter_for_pe parameter to automatically identify and trim adapter sequences in paired-end reads, primer sequence trimming with Seqtk (v1.43), and a standardized removal of 20 bp from the 5′ ends of both forward and reverse reads. Based on the Vsearch software and the Easy amplicon pipeline [41], we followed the procedure below for amplicon species annotation: Vsearch (v2.28.1) [42] was used to generate the operational taxonomic unit (OTU) table, sequences were deduplicated and retain those appear at least three times, followed by clustering the deduplicated sequences into OTUs based on 97% similarity and *de novo* chimera removal. Taxonomic composition was analyzed using QIIME2 (v2024.2.06) [43] and Vsearch sintax. OTUs were classified with two workflows: QIIME2 utilized a pretrained Greengenes classifier (gg_2022_10_backbone_full_length.nb.qza) [44], while Vsearch employed the SINTAX algorithm with the Greengenes reference (v13.5). Results were visualized using Krona [45] for an interactive exploration of taxonomic distributions. Besides, the functional prediction for each amplicon sample was performed by mapping the taxonomic data to functional pathways based on the FAPROTAX (v1.2.10) [46].

Based on the sequencing data of various species collected, we selected insect species with >10 samples and identified their core microbiome using metrics including mean abundance, standard deviation, coefficient of variation (CV), and coverage across samples. The representative abundance of each species’ core microbiome was calculated using the formula: “Representative abundance = mean abundance + coverage + (2 − cv).” The representative abundance values were normalized to reflect the relative importance of each core microbiome species. To ensure the representativeness of the core microbiome, species with a CV > 2 and those found in only one sample were filtered out. The results of the core microbiome composition were also visualized using KronaTools [45] and can be viewed on the page for each host species.

### Genome and gene catalog construction

The 3520 newly recovered MAGs, along with 2078 insect symbiont genomes from literature and public databases, including NCBI [31], NGDC [33], and the Bacterial and Viral Bioinformatics Resource Center (BV-BRC) [47], were combined and subjected to CheckM (v2.17) for quality assessment. Based on the MIMAG standard [48], the quality score (QS), defined as “completeness − 5 × contamination,” was calculated and low-quality MAGs (completeness <50% or contamination >10% or QS < 50) were removed. Taxonomic annotation was performed using the GTDB-Tk (v2.4.0) with the classify_wf function under default parameter settings (dataset r220) [49]. In addition, the taxonomic and functional annotation from literature were also incorporated.

Gene prediction was subsequently performed using Prodigal (v2.6.3) [50], operating in metagenomic mode (-p meta) for metagenome contigs and in single-genome model (-p single) for genomes. The nonredundant gene catalog for each host species was constructed from the predicted genes by CD-HIT (v4.8.1) [51] with the parameter “-c 0.9 -G 0 -M 0 -T 64 -aS 0.9” to cluster the genes with the criteria of identity ≥90% and coverage ≥90%. The gene catalog was functionally annotated using DIAMOND (v2.0.15) [52] by aligning the predicted genes against the nonredundant protein database (NR) to get the potential function description. Subsequently, eggNOG-mapper (v2.1.12) [53] with the eggNOG DB 5.0.2 dataset [54] was employed to assign Clusters of Orthologous Groups (COG) functional categories, KEGG pathways, Pfam protein domain predictions, and Enzyme Commission (EC) numbers. Pfam domains were annotated through orthology-based transfer as implemented in eggNOG-mapper, with HMMER3 applied for refinement to ensure accurate identification of domain architectures.

### Website architecture and implementation

The website architecture consists of both front-end and back-end components, each selected to optimize performance, scalability, and ease of development. For the front-end, the atomic CSS framework TailwindCSS (v3.4.10) was used to design the interface. At the backend, the Django (v4.2) framework was employed with Python (v3.9). The database was powered by PostgreSQL (v16.1), chosen for its reliability and scalability in handling large datasets. For deployment, the website was hosted on an Alibaba Cloud server with system CentOS 7.4. Gunicorn (v23.0.0) served as the WSGI server, handling application requests and communication with the web server, while Nginx (v1.20.1) functioned as a reverse proxy, managing HTTP traffic and serving static files. The interactive visualizations and charts were created using ECharts.js (v5.6.0), Three.js (v4.5), Vanta.js (v0.5.24), and Python package plotly (v5.24.1). Bioinformatic tools BLAST (v2.14.0) [55] and Diamond (v2.1.8) [52] were integrated for online analysis.

### Batch functional annotation of insect symbionts

The batch annotation tool utilizes a scoring-based algorithm to efficiently identify potential functional insect symbionts from taxonomic composition data. The tool supports multiple standard input formats, including Kraken2 reports, MetaPhlAn outputs, and Krona taxonomic data. The confidence score, used to evaluate potential functional symbionts, is derived through an integrated weighting system that combines multiple components: (i) a base score, calculated based on the abundance of the symbiont in the input data; (ii) bonuses for taxonomic and host multi-level matching; and (iii) a function score, based on function tags and the length of the function description (up to a maximum of 5 points). The matching algorithm follows a hierarchical, multi-step approach, starting with species-level taxonomic matching, where species names are standardized and exact matches are identified. If species-level matches are not found, the algorithm proceeds with genus-level matching. Similarly, the user-provided host also undergoes multi-level taxonomic comparisons. Different matching levels contribute varying bonuses to reflect the degree of alignment between the user-provided symbionts and the records in the database. For example, matches at the symbiont species or host order level each provide a bonus of 10 points. Batch annotation results are displayed in descending order based on their confidence scores, with only the top three matching records retained per symbiont.

### iSymSeek

To develop iSymSeek, we constructed a domain-specific pretraining dataset focused on insect symbionts. A total of 9560 relevant research papers were initially collected, from which 223 high-quality review and primary research articles were manually curated. Text preprocessing was conducted using pdfMiner3k (v1.3.4), which included content extraction, formatting normalization, and segmentation. Key sections of the processed text were then annotated by online Large Language Model (LLM) and subsequently extracted for conversion into a structured question–answer format following the Alpaca-style JSON schema, which was implemented through a python-based data processing pipeline. The resulting dataset serves as the foundation for the retrieval augmented response generation of the iSymSeek, enhancing its ability to provide contextually relevant and accurate academic support. Additionally, the insect symbiont records in the database were integrated into a TSV-formatted dataset, containing key structured information, including symbiont names, taxonomic classification, host species, host taxonomy, localization, transmission mode, functional description, functional classification, and supporting literature. The help documentation and a frequently asked questions (FAQ) dataset were also compiled as a structured guide for database usage.

Based on these datasets, RAGflow (v0.15.1) was selected to construct the final framework of iSymSeek. First, RAGflow’s built-in document parsing engine was used to structure the datasets into distinct knowledge bases (General, Q&A, and Table). Next, a chatbot was built upon this framework and powered by the DeepSeek API for response generation. Finally, an Agent-based framework was implemented to handle variable extraction, query classification, language conversion, and knowledge retrieval, enhancing the iSymSeek’s capacity to accurately address specialized inquiries on insect symbionts.

## Results

### Data content and statistics

Based on a systematic literature survey and large-scale sequencing data collection, we constructed iSymBase, an integrative resource of insect symbionts. The current version of iSymBase contains 2657 functional records of insect symbionts across 795 insect host species, extracted from 8009 peer-reviewed articles. Each record was annotated with 42 standardized functional tags based original function description. Besides, iSymBase incorporates 1494 metagenomes and 14 992 amplicon sequencing datasets with taxonomic and genomic annotations. A total of 3648 insect symbiont genomes are included in the iSymBase genome catalog, comprising 1570 newly reconstructed metagenome-assembled genomes (MAGs), 498 genomes curated from published studies, and 1580 genomes retrieved from publicly available databases. The database also provides a standardized gene catalog, comprising 23 041 894 nonredundant genes, functionally annotated with COG, KEGG, and Pfam annotations. Overall, by combining manually curated literature data with systematically processed genomic resources, iSymBase provides the most comprehensive collection of functional records and genomic datasets for insect symbionts (Table 2).

**Table 2:** Summary of data types and taxonomic coverage in iSymBase.

**Data type** ^**a**^	**Units**	**Number of entries**	**Host species**	**Symbiont genera**	**Symbiont species**
Functional Records	Record	2657	795	337	1122
Metagenomes	Sample	1494	164	–	–
Amplicons	Sample	14 992	728	–	–
Genomes	Assembly	3648	237	222	1788
Genes	Sequence	23 041 894	320	–	–

a All types of symbiosis-related datasets currently were integrated into iSymBase, including their respective host species and symbiont-level taxonomic annotations. “Number of entries” refers to the total number of individual records or datasets for each data type (e.g. functional records, metagenome samples, annotated gene sequences, and genome assemblies). Missing values (“–”) indicate certain taxonomic information not applicable for certain data types (e.g. metagenomes, amplicons, and gene datasets).

### Functional symbiont records and interaction network

**Figure 1:**
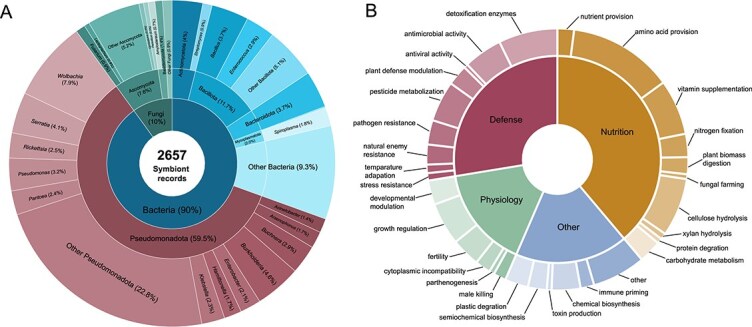
Statistics of the curated insect symbiont records. (A) Overdistribution of insect symbiont records across various taxonomic levels. (B) Distribution of functional tags for insect symbionts based on literature.

To construct a feasible dataset of functional insect symbionts, a comprehensive search was conducted across public literature databases including Web of Science, Google Scholar, and PubMed, using combinations of keywords such as “insect symbiont,” “insect gut community,” “insect endosymbiont,” and “insect microbiome,” together with related terms. The retrieved 9560 publications were manually screened to exclude those that were irrelevant to insect symbionts or those that lacked experimental functional evidence, including studies confirming the absence of a symbiont–host functional relationship those that did not provide functional validation, including studies that experimentally confirmed the absence of a symbiont–host functional relationship. Then, 8009 filtered supporting publications were included for data extraction (Supporting Information Table S2), among which 1194 provided valid functional evidence that contributed to the final dataset. In terms of taxonomy, over 89% of the recorded symbionts were bacterial, with Pseudomonadota representing the most abundant phylum, followed by Bacillota and Bacteroidota (Fig. 1A). In contrast, the fungal symbionts were predominantly from the phylum Ascomycota. The 2657 functional symbiont records encompassed insect hosts spanning 15 orders and 795 species, with Hemiptera occupying the highest proportion (30.6%) among records. Based on original functional description extracted from the literature, these symbiont records were classified into 42 categories (Fig. 1B). Analysis of the curated dataset revealed that nutrient provision, digestive enzyme activity, and reproductive manipulation are the most frequently documented symbiont functions, highlighting their ecological significance and central roles in shaping host physiology and evolutionary trajectories. To further explore host–symbiont relationships, an insect–symbiont interaction network was constructed based on host species and symbiont genus-level associations. This network uncovered nonrandom patterns of host specificity and functional clustering, offering insights into the distribution of symbiont-mediated ecological traits across insect lineages.

### Genome and gene catalog

We developed the first comprehensive genome and gene catalogs for insect symbionts, integrating 3648 high-quality genomes and 23 041 894 nonredundant genes, establishing a foundational resource for insect symbiont research, inspired by principles from human and environmental microbiome research [56–58]. The genome catalog comprises 1570 newly reconstructed metagenome-assembled genomes (MAGs) derived from 1494 manually curated symbiont-related metagenomes, 1580 genomes sourced from public databases (NCBI, NGDC, and BV-BRC), and 498 genomes extracted from published literature. After quality assessment using CheckM, low-quality MAGs (completeness <50%, contamination >10%, or QS <50) were filtered out, resulting in a final dataset of high-quality genomes. Taxonomic annotation of these genomes, performed with GTDB-Tk and cross-referencing with academic literature, identified a diverse range of microbial lineages, encompassing 222 symbiont genera and 1788 symbiont species. While the present genome catalog represents symbionts from 237 insect host species, its value and impact will continue to grow as research expands the known diversity of both insect hosts and their microbial partners as an increasingly comprehensive framework for insect symbiont research.

For gene catalog, we constructed a comprehensive, nonredundant dataset comprising 23 041 894 genes, providing an extensive resource for functional exploration of insect symbionts. Genes were predicted using Prodigal and subsequently clustered at 90% sequence identity and 90% coverage with CD-HIT, resulting in a refined, nonredundant catalog optimized for comparative analyses across insect hosts. Functional annotation of the gene catalog, performed with DIAMOND, provided insights into potential metabolic capabilities by aligning predicted genes against the NR database. The most frequently identified function included reverse transcriptase, transposase, and Major Facilitator Superfamily transporter (MFS transporter), highlighting the prevalence of mobile genetic elements and transport systems in insect symbionts. To further characterize functional traits, eggNOG-mapper was used for COG classification and KEGG pathway mapping, Pfam domain annotation, and EC number assignment, highlighting key symbiont-associated functions. Prominent KEGG annotations included biosynthesis of amino acids (ko01230), ABC transporters (ko02010), carbon metabolism (ko01200), and purine metabolism (ko00230), reflecting core metabolic and transport processes crucial for insect–symbiont interactions.

### Web interface

**Figure 2:**
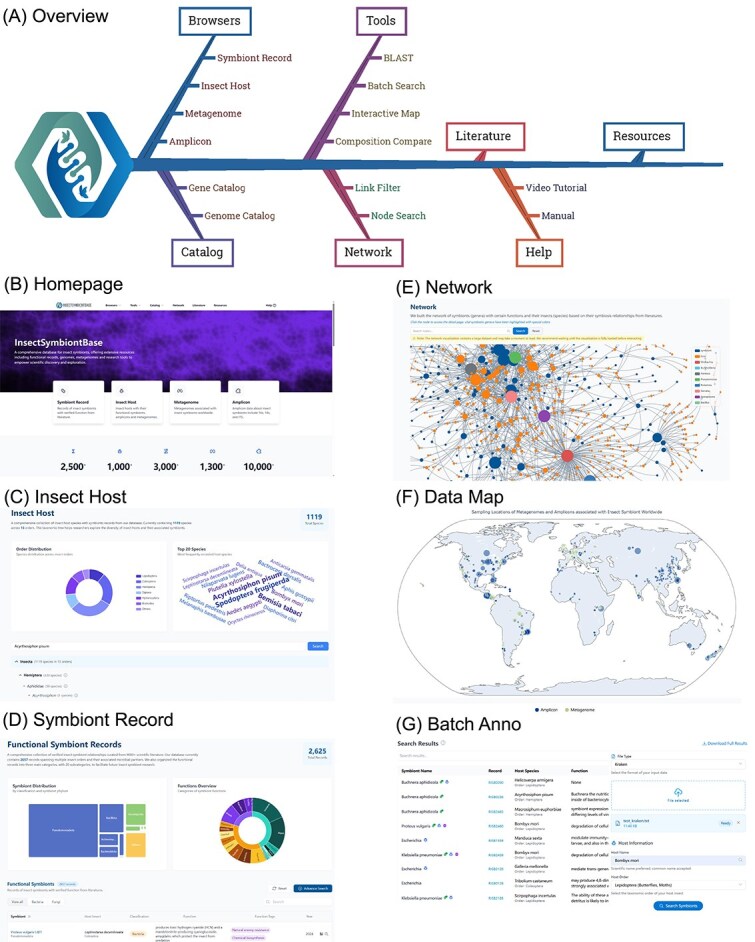
Interface overview and key pages of iSymBase. (A) Interface overview: The database offers extensive pages for browsing, analyzing, and visualizing insect symbiont data, along with detailed help documentation and external resource links. (B) Homepage: provides quick navigation, statistical summaries, update news, and the introduction of key features. (C) Insect Host: organizes hosts with integrated symbiont data in an insect phylogenetic tree. (D) Symbiont Record: provides insect symbiont functional records verified by literature. (E) Network: insect–symbiont interactive network with search and filter function. (F) Data Map: the geographical distribution of insect symbiont–related sequencing data worldwide. (G) Batch Anno: to efficiently annotate potential functional insect symbionts from uploaded taxonomic composition data.

The iSymBase database is freely accessible through a user-friendly website (http://symbiont.insect-genome.com), providing researchers with comprehensive resources on insect symbionts. The web interface allows users to browse and query detailed information on functional records, genomes, and metagenomes of insect symbionts. In addition to offering downloadable data, the platform also provides various bioinformatics tools and advanced search functionalities to facilitate scientific exploration and discovery in the field of insect symbionts (Fig. 2).

The Symbiont Record module allows users to browse insect symbiont records, including symbiont names, host species, and functional descriptions, with links to detailed reports featuring taxonomic and ecological information. The Genome and Gene Catalog modules provide genome sequences and functional annotations for insect symbionts, including annotations like GO terms, KEGG pathways, and Pfam domains, enabling researchers to explore the functional diversity of these symbionts. The Metagenome and Amplicon modules offer sequencing data with details on platforms, taxonomic classifications, and functional annotations. Interactive visualizations and downloadable data allow further exploration. iSymBase also provides cutting-edge tools, including an insect–symbiont interaction network for ecological relationship mapping, a batch annotation tool for functional symbiont detection, and the iSymSeek AI chatbot to assist with queries. The platform’s search function and intuitive navigation features ensure an efficient and smooth user experience, making it easy to find and explore relevant data across the database.

### Data access and usage

#### Basic data search

Through the basic search bar available on each webpage, users can search for symbionts, hosts, symbiont function, and related genomic resources of interest within the database. Due to the complex attributes of the symbiont records, the auto-completion feature was supported in the search function, which allows more user-friendly and faster queries. After entering the first few letters, the search bar will display predictive search suggestions based on fields such as symbiont, host, function, and function tags from the database. Additionally, each webpage provides an advanced multi-field search feature, allowing users to perform precise data queries. As for sequences, users can use the Basic Local Alignment Search Tool (BLAST) sequence similarity search to find target insect symbiont or its genes.

#### Host-based and phylogenetic query

iSymBase also provides a range of diverse query methods to accommodate the varied data retrieval needs for insect symbiont data. First, a host-based data query system has been implemented, enabling users to efficiently access symbiont records, amplicons, metagenomes, and related literature for each host species through a one-stop query. Next, users can explore all insect species via a phylogenetic tree, which facilitates comparisons of symbiont distributions across closely related insects.

#### Exploring insect–symbiont relationships through interaction network

To provide users a tool to quickly explore the symbiosis relationships between symbionts of interest and its potential insect hosts, or between the symbionts themselves, the insect–symbiont interactive network was constructed to facilitate the systematic exploration of insect–symbiont relationships through multi-layered query approaches (Fig. 2E). Users can query nodes representing either symbiont genera or insect hosts, with hover interactions revealing ecological connections via linked edges. The integrated search function supports taxonomic queries (genus/species names), visually highlighting matched nodes with a golden halo while maintaining the overall network context. Advanced filtering options allow users to apply combined constraints on host taxonomy and symbiont functional attributes, enabling the isolation of specific relationship subsets. Additionally, dynamic node dragging and zooming capabilities help users identify topological patterns, while direct access to related literature through edge clicks fosters hypothesis generation concerning co-evolutionary relationships and functional niche specialization.

#### Batch functional annotation of insect symbionts

To help users rapidly pinpoint putative functional symbionts in their own sequencing results, we developed a batch functional annotation tool that annotates insect-associated taxa in uploaded community-profiling files (Fig. 2G). The tool accepts common community-profiling formats such as Kraken2 reports, MetaPhlAn outputs, and Krona input tables, then performs large scale matching against the curated symbiont records in our database. During this process, prokaryotic branches at the genus or species level are systematically mapped to symbiont functions that have already been established from functional records extracted from the database and their supporting literature. Each matched taxon receives a relevance score that highlights candidates with plausible functional roles, and every candidate links directly to its underlying raw record and the cited studies, enabling users to inspect detailed annotations and explore the evidence base.

### Additional functionalities

iSymBase integrates iSymSeek, an AI-powered chatbot based on the DeepSeek language model, to assist researchers in navigating the database and exploring insect symbiont knowledge. Powered by retrieval-augmented generation (RAG), iSymSeek draws on structured database tables and curated literature to deliver context-aware responses. A dedicated agent further enhances its ability to provide accurate dataset retrieval, functional interpretation, and academic support for symbiont-related research.

iSymBase features an interactive data submission portal that enables researchers to contribute their own newly published datasets on insect symbionts. In addition to user submissions, the database is continuously updated with curated records from the latest scientific literature, ensuring its relevance and completeness. This community-driven model fosters an open and collaborative research environment, encouraging shared contributions and continuous refinement. All datasets in iSymBase are freely accessible through the download page, and users are encouraged to cite original sources to acknowledge authorship and maintain scientific integrity.

### Case study: Identification of potential functional symbionts in the gut microbiota of *Chilo suppressalis*

*Chilo suppressalis* (the rice striped stem borer) is one of the most destructive pests of rice in Asia. Its larvae bore into rice stems, causing “dead hearts” and “whiteheads,” which lead to severe yield losses each year [59, 60]. Studies show that the gut microbiota of *C. suppressalis* varies across regions and diets, with certain bacteria involved in detoxification and plant defense modulation, suggesting roles in host adaptation [61, 62]. However, despite a few studies, most gut symbionts remain poorly characterized in terms of function. In this case study, we used iSymBase to identify and annotate potential functional symbionts in the gut microbiota of *C. suppressalis*, illustrating how the database can facilitate functional screening and hypothesis generation for insect–microbe interaction studies.

**Figure 3:**
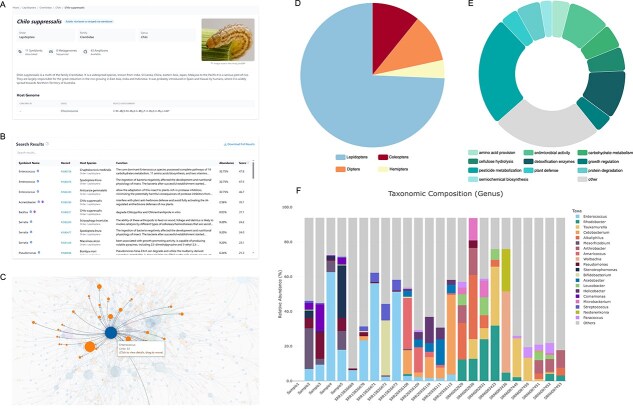
Case study: Identification of potential functional symbionts in the gut microbiota of *C. suppressalis*. (A) Overview of *C. suppressalis* in iSymBase. (B) Batch functional annotation of the gut microbiota of *C. suppressalis* using the Batch Anno module. (C) Host–symbiont interaction network centered on *Enterococcus*. (D) Distribution of insect host orders associated with functional symbiont records of *Enterococcus*. (E) Distribution of function tags associated with functional symbiont records of *Enterococcus*. (F) Comparative genus-level composition of the gut microbiota of *C. suppressalis* using the Composition Compare module.

First, we examined the *C. suppressalis* symbiont profile in the Insect Host module of iSymBase, which contains 15 curated symbiont records and 43 associated amplicon datasets, along with symbiont data from six closely related species within the same family (Fig. 3A). Using our self-generated amplicon datasets of *C. suppressalis* (Supplementary Table S3), we applied the Batch Anno module in iSymBase to conduct online prediction of potential functional symbionts. By integrating the prediction scores with relative abundance data, *Enterococcus* emerged as the most probable functional symbiont within the gut microbiota of *C. suppressalis*, with matched records primarily related to nutrient provision and protein digestion (Fig. 3B). In addition, the prediction results also annotated two other bacterial genera, *Acinetobacter* and *Bacillus*, both of which have been previously verified as functional symbionts in *C. suppressalis*, involved in regulating plant defense and pesticide degradation. Given that *Enterococcus* has not yet been functionally characterized in this host, we focused subsequent analyses on its potential ecological functions.

Within the host–symbiont interaction network, *Enterococcus* displayed strong associations with multiple insect hosts, among which *Spodoptera frugiperda* (fall armyworm) showed the most frequent co-occurrence and functional connections (Fig. 3C). Subsequently, we retrieved 76 *Enterococcus* records from the Symbiont Record module. Of these, 72.37% were linked to Lepidopteran hosts, suggesting a strong functional association between *Enterococcus* and Lepidoptera insects (Fig. 3D). Most functional records were related to pesticide metabolization and detoxification, highlighting the potential contribution of *Enterococcus* to host metabolic adaptation (Fig. 3E). To complement the functional annotation results, we examined the gut microbial composition of *C. suppressalis* through the Composition Compare module. The community was relatively stable at the phylum level, dominated by Proteobacteria and Firmicutes, but showed marked genus-level variation associated with dietary and pesticide treatments (Fig. 3F). To support subsequent strain-level analysis, we further accessed 72 *Enterococcus* genomes from the Genome Catalog, of which 12 were obtained from published literature, establishing a genomic foundation for exploring the functional potential of *Enterococcus* in *C. suppressalis*. Overall, this case study demonstrates the utility of iSymBase as an integrative platform for linking symbiont diversity, function, and genomics, thereby facilitating hypothesis-driven research on insect–symbiont ecology.

## Discussion

The surge in publications on insect symbionts has highlighted the need for specialized tools to systematically categorize and organize the massive data, facilitating deeper analyses and maximizing its potential for insights. Existing web resources have documented the microbial genomes alongside their symbiotic relationships [25, 27, 47, 63, 64]. However, these resources primarily serve as repositories for microbial genomic data, offering only limited documentation of insect–symbiont relationships and lacking detailed functional information. Due to the absence of the genome for majority of functional symbionts in literature, researchers face difficulties in obtaining a comprehensive understanding of insect–symbiont interactions from existing resources.

In this study, we systematically extracted and curated detailed functional records of insect symbionts, along with their genomic resources from the academic literature, laying the foundation for the construction of iSymBase. Through the intuitive and well-structured web interface, users can easily get access to an extensive array of records and genomic data related to insect symbionts through iSymBase, without exhaustive searches for insect symbiont functional information across multiple literature and databases. Beyond data query, the database offers a range of online tools for users to analyze their insect symbiont datasets, such as a batch annotation tool designed to efficiently identify potential functional insect symbionts from uploaded taxonomic composition data. To our knowledge, iSymBase is the first manually curated database for insect symbionts that offers a standardized classification of functional records and genomic data resources. With manually curated records that extend past traditional model systems, iSymBase enables comprehensive analyses of insect–symbiont dynamics, drives the exploration of new symbionts, and provides a scientific data foundation for sustainable potential applications, such as microbiome-based pest control strategies and engineering [65–67]. In addition, the database provides classified functional symbiont records with corresponding genomes, which can serve as foundational datasets for future studies on machine learning or large language model–based functional predictions of insect symbionts.

iSymBase still has certain limitations. First, all functional records documented in the database lack specific details on transmission modes and localization, primarily because such information is often absent from the original literature. Meanwhile, some viruses that is not currently documented exhibit complex interactions with insect symbionts, representing an important yet underrepresented aspect of insect–symbiont relationships. To address these limitations, ongoing efforts will focus on compiling and curating additional data, thereby improving the coverage and accessibility of future iSymBase updates. Additionally, the identification of potential symbionts is currently based on the strong but imperfect link between symbiont phylogeny and biomolecular function, lacking the integration of symbiont genomic data due to the scarcity of functionally annotated symbiont genomes. An increasing number of studies have explored the relationship between insect symbiont genomes and their functions [68–70], suggesting that machine learning and LLM-based functional prediction for insect symbionts will be a key direction for future research. With the rapid accumulation of diverse insect symbiont genomic data, we are responsible for frequently updating datasets in the database and implementing new online functionalities for insect symbionts.

In summary, iSymBase documents 2657 functional records of insect symbionts, spanning >795 host species, with verified experimental evidence and supporting reference. Beyond functional records, the database compiles a comprehensive repository of genomic data, including 1494 metagenomes, 14 992 amplicon datasets, together with standardized genome and gene catalogs, providing a critical foundation for studying insect symbiont diversity, evolution, and functional potential. iSymBase serves as a user-friendly and practical platform that enables users to efficiently browse, query, visualize, and download the functional records and genomic data, which can be used to foster the future research into the novel symbionts discovery and functional mechanism elucidation.

## Supplementary Material

Supporting_Information_Table_ycag128

## Data Availability

The datasets generated during the current study are available in the iSymBase repository (http://symbiont.insect-genome.com). The analysis workflow and related software parameters are available in the GitHub repository (https://github.com/Wzuoqi/iSymBase).
